# Serum, urinary, and salivary nitric oxide in rheumatoid arthritis: complexities of interpreting nitric oxide measures

**DOI:** 10.1186/ar2030

**Published:** 2006-08-14

**Authors:** J Brice Weinberg, Thomas Lang, William E Wilkinson, David S Pisetsky, E William St Clair

**Affiliations:** 1 Veterans Affairs Medical Center, 508 Fulton Street, Durham, NC 27705, USA; 2 Duke University Medical Center, 508 Fulton Street, Durham, NC 27705, USA; 3 University of Maryland School of Medicine, 10 South Pine Street, Baltimore, MD, USA 21201

## Abstract

Nitric oxide (NO) may play important roles in rheumatoid arthritis (RA). RA is an inflammatory disease involving joints and other systems including salivary glands. To assess NO production in RA patients, we compared levels of serum, urine, and salivary nitrite and nitrate (NOx) in patients with RA and normal subjects, and we examined the relationships of these measures to disease activity. Serum, urine, and NOx levels as well as renal creatinine, NOx clearance and fractional excretion rates were compared in 25 RA patients and 20 age- and gender-matched healthy controls. Subjects were hospitalized for 3 days and placed on a NOxrestricted diet. NOx was assayed using nitrate reductase and the Griess reagent. RA activity was assessed using standard clinical and laboratory measures. While consuming a restricted diet for 3 days to eliminate the effects of oral intake of NOx, 24 hour urinary NOx excretion decreased in both RA patients and healthy controls. Urine NOx levels at all time points were not significantly different between RA patients and normal subjects. Serum NOx levels also decreased during the 3 days of NOx restriction, but RA patients had higher serum NOx levels at all time points compared with the control group. Likewise, serum NOx/creatinine ratios were higher in RA patients than in controls. Although basal salivary flow rate and tear flow were lower in RA patients, salivary NOx levels did not differ between normal and RA subjects. While renal creatinine clearance was not different between the two groups, we found that RA patients had lower renal NOx clearance and lower renal NOx fractional excretion. After correction of *p *values for multiple comparisons, there were no significant relationships for the RA group between measures of disease activity and the urinary NOx, serum NOx, or urinary NOx clearance. Despite interest in the use of NO as a marker of disease activity, alterations in renal NOx clearance and fractional excretion in RA make it difficult to assess *in vivo *NO production even with strict dietary restriction of NOx intake.

## Introduction

Nitric oxide (NO) is an important mediator of diverse physiologic and pathologic processes, including arthritis [1,2]. Joint inflammation in autoimmune MRL-*lpr*/*lpr *mice and rats with adjuvant-induced arthritis [3-9] is dependent on the enhanced production of NO. NO, a lipid- and water-soluble gas, is ideally suited as a potent inflammatory mediator because of its strong reactivity with oxygen, superoxide, and iron-containing compounds. This inherent reactivity of NO translates into a relatively short half-life (for example 1 to 10 s), which has made it technically difficult to quantify in solution. Instead of directly measuring NO, investigators have estimated NO production by measuring levels of nitrate (NO_3_^-^) and nitrite (NO_2_^-^), stable anions derived from the reaction of NO with superoxide. In general, serum levels and urinary excretion of nitrite + nitrate (NOx) reflect the total production of NO by the body [10,11]. Care must be taken in the interpretation of results from these studies, because ingested nitrite or nitrate and renal insufficiency elevate both serum and urine nitrate as well as nitrite [10,12,13].

Although previous work has provided evidence in rheumatoid arthritis (RA) for increased production of systemic NO [14-21] and increased expression of inducible NO synthase (NOS2) and production of NO [22], most studies of urine and serum NOx levels have been performed in patients eating a normal diet [17] or after only an overnight fast [15]. Other approaches that assess NO production are less subject to dietary influences. For example, nitrotyrosines, which are formed from the reaction of peroxynitrite (a product of NO and superoxide) with tyrosine, can be measured by immunoassay or high-performance liquid chromatography [16,23]. Using this method, Kaur and Halliwell [16] have detected nitrotyrosines in serum and synovial fluid from patients with active RA, but not in serum from controls.

In the present study, we assessed NO production *in vivo *by measuring levels of NOx in urine, serum, and saliva in patients with active RA and in normal subjects under conditions of strict dietary NOx restriction. In a comparison between patients with RA and normal subjects, we found that patients with RA had comparable levels of NOx in urine and saliva, elevated serum NOx and serum NOx/creatinine, normal renal creatinine clearance, and reduced renal NOx clearance and fractional excretion. The reduced renal NOx clearance and fractional excretion limit the use of serum NOx and urine NOx excretion as parameters of NO production in patients with RA and their potential as disease markers.

## Materials and methods

### Patients and controls

Twenty-five patients who met the American College of Rheumatology 1987 revised criteria [24] for the classification of RA were recruited from the Duke University Medical Center (DUMC) Rheumatology Outpatient Clinics. The patients were taking stable doses of prednisone (not more than 10 mg/day) and nonsteroidal anti-inflammatory drugs (NSAIDs) for at least 2 weeks before study entry. If they were taking second-line drugs, such as methotrexate, hydroxychloroquine, gold, sulfasalazine, or azathioprine, doses of these medications were stable for at least 4 weeks before study entry. No subjects were taking anti-cytokine agents such as anti-TNF antibody, a treatment that we have shown decreases the overexpression of blood mononuclear cell NOS2 in RA [25]. For comparison, 20 age-matched (within 5 years) and gender-matched subjects without RA were recruited by newspaper advertisement. Patients and controls who had coexisting chronic inflammatory conditions, active infections, malignancy, cirrhosis, or a serum creatinine level of more than 2.5 mg/dl were excluded from participation. Patients were not allowed nitrate-containing medications or permitted to smoke during the study period. The DUMC Institutional Review Board approved the protocol, and informed consent was obtained from each subject before participation. These are the same patient and control subjects as those reported previously in whom we showed greater NOS2 expression and *in vitro *NO production in the patients with RA than in controls [22].

### Study design

Eligible subjects were hospitalized for 3 days on the inpatient unit of the General Clinical Research Center at DUMC. Subjects had a complete history and physical examination at baseline to confirm eligibility. In addition, the patients with RA were comprehensively evaluated for disease activity with the use of the following measures:

1. Tender and swollen joint count (maximum of 68 tender and 66 swollen joints).

2. Duration of morning stiffness.

3. Patient assessment of pain on a 10 cm visual analog scale.

4. Physician global assessment of disease activity with the use of a 10 cm analog scale.

5. Functional capacity determined with the modified Stanford Health Assessment Questionnaire (mHAQ) [26].

The functional class was determined with the American College of Rheumatology 1991 revised criteria for the classification of global functional status [27]. Rheumatologic assessments and routine laboratory studies were performed at baseline and on day 3. Because no significant differences in disease measures were found between baseline and day 3, the disease-related parameters at baseline were used in the analysis below.

The characteristics of the patients with RA and the healthy controls have been published previously [22]. The two groups were similar with respect to median age (patients with RA, 58 years; controls, 56 years) and gender (patients with RA, 17 women and 8 men; controls, 14 women and 6 men). The median duration of disease for the patients with RA was 9 years. In general, the patients with RA had severe disease, as reflected by the high proportion of patients with subcutaneous nodules (48%), the high proportion of patients currently taking a second-line drug (72%), and the frequent past use of second-line drugs (72%). Disease activity in the RA group was characterized by high median tender (31) and swollen (28) joint counts, prolonged morning stiffness (median duration 60 minutes), a median erythrocyte sedimentation rate of 26 mm/hour, a median CRP of 13 mg/l, and moderate functional disability (median mHAQ score 1.62). No control subjects were taking prednisone, and only two controls were taking a NSAID at the time of the study.

### Dietary intervention

The subjects were fed a low-NOx diet that met the recommended allowances for weight maintenance in kilocalories, carbohydrate, fat, and protein. Two caloric diets were available for selection by subjects: first, a 2,000 kcal diet of 18% protein, 60% carbohydrates, and 22% fat, yielding about 100 μmoles of NOx per day; and second, a 2,500 kcal diet of 19% protein, 60% carbohydrates, and 21% fat, yielding about 110 μmoles of NOx per day. The amount of NOx in these diets was determined as described previously [28]. The diet allowed unlimited consumption of distilled water. A research dietician obtained a dietary history from each subject at the time of admission to estimate the oral intake of NOx during the previous 24 hours and also monitored the daily oral intake of food and beverages in the hospital to estimate the daily consumption of NOx.

### Blood, urine, and saliva collection, and NOx assays

Blood samples were drawn by venipuncture from subjects at 0, 24, 48, and 72 hours for determination of serum creatinine and NOx concentrations. NO reacts with oxygen to form nitrite and nitrate. We used the Griess reagent to measure nitrite [29,30]. We first converted all nitrate to nitrite with bacterial nitrate reductase, and then this nitrite (representing total nitrite plus nitrate [NOx]) was measured with the Griess reagent. Propan-2-ol ('isopropanol') was added to urine collected during a 24 hour period at a ratio of 1:10 (propan-2-ol/urine) to prevent bacterial growth, because contaminating bacterial enzymes can introduce errors. Urine and saliva were used unprocessed, but serum samples were filtered with Centricon filters (Amicon, Beverly, MA, USA) before performing the NOx assays. We incubated 50 μl of sample for 30 minutes with 7 μl of 1 M Tris pH 7.5, 10 μl of 0.02 mM NADPH, 20 μl of 5 mM glucose 6-phosphate (G6P), 3 μl of 10 units/ml glucose-6-phosphate dehydrogenase (G6PD), and 10 μl of a 10 unit/mL solution of nitrate reductase at room temperature (20 to 23°C). The G6P and G6PD were used to eliminate excess NADPH, which might interfere with the overall Griess reaction. We incubated 75 μl of this mixture for 10 minutes with 75 μl of Griess reagent I (3% sulfanilamide in 2.5% phosphoric acid) and Griess reagent II (0.3% naphthylethylenediamine in 2.5% phosphoric acid) at room temperature. The absorbances at 550 nm were measured, and concentrations of NOx were determined by comparison with a standard curve generated with reagent nitrate.

Twenty-four-hour urine samples were collected on day 1 (0 to 24 hours), day 2 (24 to 48 hours), and day 3 (48 to 72 hours) to quantify urinary excretion of NOx. Urine volume and creatinine content were measured for each 24-hour sample to calculate creatinine and NOx clearances. The renal clearance of NOx was calculated by the following formula: clearance = (Urine flow rate (liters/24 hours) × Urine NOx (mol/l))/(Serum NOx (mol/l)). Renal fractional excretion of NOx was determined by dividing the renal NOx clearance by the creatinine clearance. Saliva specimens were obtained after 72 hours for measurement of basal and stimulated salivary NO production with the use of a method described previously [31]. Subjects first rinsed their mouth with an antiseptic (Peridex) to reduce bacterial contamination. For basal measurements, 0.2 to 0.3 ml of saliva was collected within 1 minute of the antiseptic rinse. For measurement of stimulated flow, subjects rinsed their mouth with 1 ml of lemon juice followed by a second rinse with distilled water. Samples (0.2 to 0.3 ml) were collected at 0 to 1 minute and at 5 to 6 minutes. The Schirmer's tear test was done without anesthesia as noted previously [32].

### Analyses

Descriptive statistics were expressed in terms of the median and interquartile range for continuous variables. Comparisons between cases and controls regarding various parameters of NOx, NOS, and disease measures were made using the Wilcoxon rank sum test. The comparisons of renal clearance and fractional excretion of NOx between cases and controls in each of the three 24-hour time periods used only the 23 RA patients and 17 controls for whom assay results were available for the first two time periods. For the 8 RA patients with missing observations for the third 24-hour period, it was conservatively assumed that their NOx clearance and fractional excretion on day 3 were the same as that on day 2 since the 15 other RA patients all had lower values on day 3 for both of these measures. The relationship of urinary NOx to disease measures was determined using a Spearman correlation coefficient.

## Results

### Dietary intake of NOx

The subjects were hospitalized and placed on a NOx-restricted diet to minimize exogenous sources of these constituents. The pre-study dietary content of NOx was highly variable between individuals (Table 1), but normal subjects were not significantly different from patients with RA. Once individuals had been placed on the restricted diet, we confirmed by careful history and inspection of food trays that they had consumed only the minimal amounts of NOx allowed by the diet. Although control subjects on days 2 and 3 ingested slightly more NOx than RA subjects did, these differences were very small in comparison with the measured levels of these compounds in the serum and urine.

### Urine and serum NOx levels

**Figure 1:**
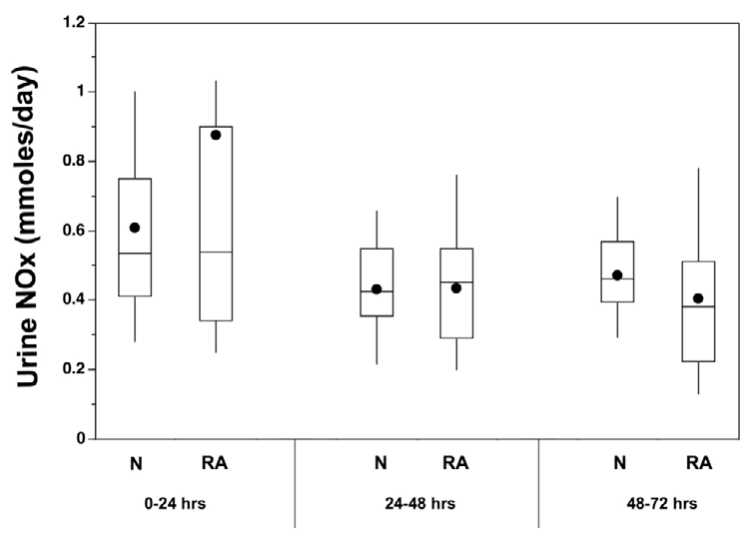
Urinary excretion of NOx. Consecutive 24-hour urine samples were collected from 25 patients with active rheumatoid arthritis (RA) and 20 healthy controls (N) who were receiving a diet low in nitrate and nitrite. During each of the time periods, no statistically significant differences were observed between the two groups in 24-hour urinary excretion of NOx. Values are expressed as medians (horizontal lines), means (filled circles), interquartile ranges (boxes), and 10th to 90th centile ranges (whiskers). NOx, NO_2_^- ^(nitrite) + NO_3_^- ^(nitrate).

**Figure 2:**
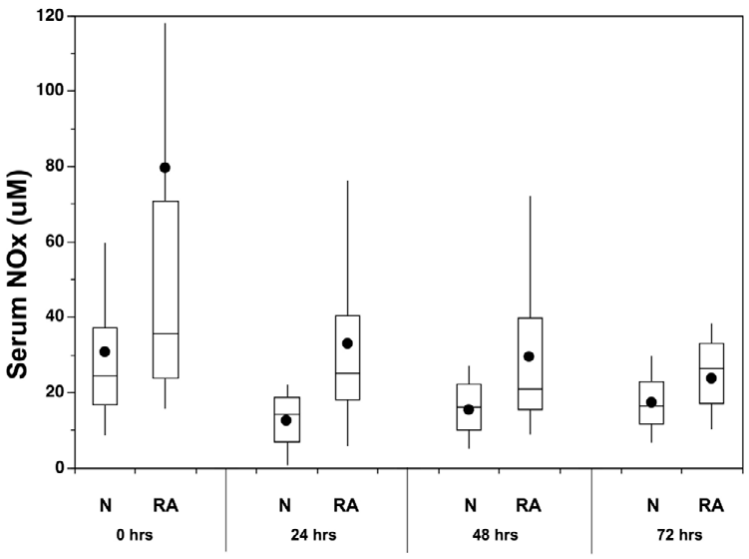
Serum concentration of NOx. Serum was obtained from 25 patients with active rheumatoid arthritis (RA) and 20 healthy controls (N) at 0, 24, 48, and 72 hours after the initiation of a diet low in nitrate and nitrite. Serum NOx concentrations were higher in the RA group than in the control group at 0, 24, 48, and 72 hours (*p *= 0.028, 0.0004, 0.046, and 0.053, respectively). Values are expressed as medians (horizontal lines), means (filled circles), interquartile ranges (boxes), and 10th to 90th centile ranges (whiskers). NOx, NO_2_^- ^(nitrite) + NO_3_^- ^(nitrate).

**Figure 3:**
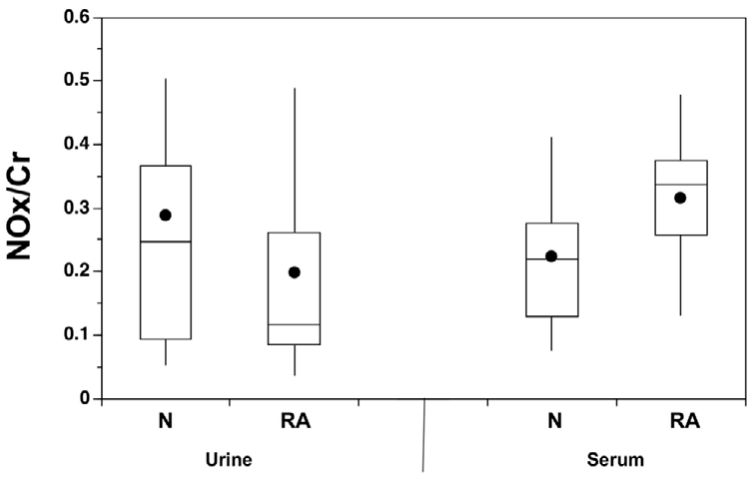
NOx/creatinine ratios in urine and serum. Urine and serum samples from 25 patients with active rheumatoid arthritis (RA) and 20 healthy controls (N) were analyzed 72 hours after initiation of a diet low in nitrate and nitrite. NOx/creatinine ratios were calculated and plotted. The difference in serum NOx/creatinine values between the RA and control groups was statistically significant (*p *= 0.013). The difference between control and RA subjects for urine NOx/creatinine was not statistically significant. NOx, NO_2_^- ^(nitrite) + NO_3_^- ^(nitrate).

In conditions of low ingestion of exogenous NOx and normal function, urinary NOx excretion reflects total production of NO by the body [10,33]. On the basis of other studies [10,12,33], we predicted that our dietary intervention would quickly decrease urinary NOx excretion, which would then stabilize after about 24 to 48 hours. This prediction was confirmed in healthy controls and patients with RA, whose urinary NOx excretions decreased by 21 to 30% over the 72-hour interval (Figure 1). The differences at each period of urine collection were not different when comparing normal controls with patients with RA (*p *> 0.05). Serum NOx levels decreased to an even greater degree with dietary NOx restriction (as much as 42% decrease) and stabilized over 72 hours (Figure 2). Despite an absence of increased urinary NOx excretion in patients with RA, patients with RA had significantly higher serum NOx levels at all time points (Figure 2). Likewise, day 3 serum NOx/creatinine ratios were significantly higher in patients with RA than in controls, whereas urinary NOx/creatinine ratios were not significantly different between these two groups (Figure 3). The use of NOx/creatinine ratios acts as a control for individual differences in creatinine clearances. The finding of increased NOx and NOx/creatinine ratios in serum (but not in urine) in patients with RA raised the possibility that controls and subjects with RA might differ in their renal elimination of NOx.

### Renal clearance and fractional excretion of NOx

The creatinine clearance was slightly higher in patients with RA, but this difference was not statistically significant (Table 2). This finding indicates that a reduction in glomerular filtration rate in patients with RA was not a likely explanation for any elevated serum and urine NOx levels in RA. However, the renal clearance and fractional excretion of NOx were significantly lower in subjects with RA than in controls at all time points. There was day-to-day variability in the renal clearance of NOx, with lower clearances at late time points as the NOx-restricted diet diminished the total NOx load.

We performed a subgroup analysis of the patients with RA to investigate whether certain medications might be associated with altered renal clearance of NOx. The median urinary NOx clearance was higher in NSAID users (*n *= 13) than in non-users (*n *= 12) (49.8 ml/min versus 23.9 ml/min, respectively; *p *= 0.017). There were no significant differences in urinary NOx clearance between prednisone users (*n *= 16) and non-users (*n *= 9) (38.7 ml/min versus 53.1 ml/min, respectively) and methotrexate users and non-users (33.8 ml/min versus 40.9 ml/min, respectively). Our data indicate that anti-rheumatic medications were not likely to be responsible for the lower renal clearance of NOx in the patients with RA. However, it is possible that an analysis of larger numbers of subjects might show differences.

### Saliva and tears

Saliva can contain large amounts of NO catabolites [13,34]. Dietary nitrate is converted to nitrite in saliva, is swallowed, and then may appear as nitrate or nitrite in saliva (the enterosalivary circulation), with salivary NOx sometimes reaching millimolar concentrations [13,34]. Salivary flow is regulated in part by NO [31]. The prevalence of sicca symptoms in RA ranges from 25% to 65% [35-39]. We examined basal and stimulated salivary flow and basal tear flow (Schirmer's tear test), and we measured NOx levels in saliva (Table 3). Unstimulated and stimulated salivary flow rates were significantly lower in subjects with RA than in controls. Similarly, Schirmer's tear test demonstrated that patients with RA had a lower tear flow than healthy subjects. We did not measure NOx levels in tears. Basal and stimulated salivary NOx levels were not different between normal controls and patients with RA (Table 3).

### Relationship of NO parameters to disease activity

In an earlier report of studies involving these subjects, we showed that the NOS activity of freshly isolated peripheral blood mononuclear cells (PBMCs) was significantly higher in subjects with RA and that PBMCs produced more NOx *in vitro *in the basal state and after stimulation with IFN-γ [22]. Furthermore, NOS activity correlated significantly with the number of swollen and tender joints. We had postulated that other measures of NO production (serum NOx, urine NOx, serum NOx/creatinine ratios, urine NOx/creatinine ratios) would correlate with certain disease activity measures. We therefore assessed the relationships of the serum NOx levels at 72 hours, the urine NOx excretion during the period from 48 to 72 hours, the serum and urine NOx/creatinine ratios during the period from 48 to 72 hours, and NOx urinary clearance and fractional excretion during the period from 48 to 72 hours, relative to 10 RA activity parameters (erythrocyte sedimentation rate, serum C-reactive protein, hemoglobin concentration, duration of morning stiffness, physician assessment of pain (visual analog scale), patient pain self-assessment (visual analog scale), patient self-assessment of disease severity (visual analog scale), number of tender joints, number of swollen joints, and functional disability (mHAQ)). Results showed a significant correlation of the serum NOx/creatinine ratio with the number of painful joints (*r *= 0.59; *p *= 0.012), physician assessment of pain (*r *= 0.40; *p *= 0.015), and hemoglobin concentration (*r *= 0.36; *p *= 0.030). The urine NOx/creatinine ratio was significantly correlated with the mHAQ (*r *= 0.40; *p *= 0.047). After corrections for multiple comparisons, none of these remained significant. Urine NOx clearance and fractional excretion did not correlate significantly with any of the parameters. We conclude that (despite our carefully limiting the dietary intake of nitrates and nitrites) serum and urinary measures of NO production are of limited usefulness as biomarkers of disease activity in RA. It is possible that stronger correlations might be found by analyzing larger numbers of subjects.

## Discussion

NO is synthesized from L-arginine by a family of enzymes known as nitric oxide synthases. These enzymes are encoded by three separate genes and are NOS1 (neural NOS), NOS2 (inducible NOS), and NOS3 (endothelial NOS). NOS1 and NOS3 are calcium-dependent and generally produce low levels of NO involved in normal physiologic processes. In contrast, NOS type 2 is calcium-independent. Its expression is upregulated by IFNα, IFNγ, IL-1, and TNF-α as well as other pro-inflammatory mediators, resulting in sustained and high-level NO output [40,41].

Several lines of evidence implicate NO in the pathogenesis of joint inflammation. Rodents in animal models of arthritis generate abundant quantities of NO, as reflected in high levels of serum and urinary NOx that develop in association with disease manifestations [3-6,42]. Treatment of these animals with NOS inhibitors or NO quenchers suppresses NO production and effectively abrogates joint inflammation. These findings have prompted studies in humans to determine whether patients with RA, like the rodents with arthritis, also have heightened NO production. Some investigators have analyzed NO production and NOS in human synovial tissue. For example, Sakurai and colleagues [43] showed that macrophages and endothelial cells from synovial tissue of patients with RA express NOS2 mRNA and protein, and generate NO *in vitro*. We also noted that circulating mononuclear cells from patients with RA are activated to express NOS type 2 and overproduce NO [22].

Other approaches have focused on systemic NO production. In one study, Grabowski and colleagues [15] showed that patients with RA have threefold higher urinary nitrate/creatinine ratios than controls, implying that urinary NOx can be used in this clinical setting as a reliable index of excessive NO production. Farrell and colleagues found that patients with RA or osteoarthritis (OA) had higher serum nitrite (not nitrite + nitrate) levels than normal controls (with RA being higher than OA) [17]. Similarly, Euki and colleagues found that serum nitrite was higher in patients with RA than in normal controls and patients with OA, and that nitrite levels were correlated with clinical parameters of RA activity, C-reactive protein, serum TNF, and serum IL-6 [14]. These workers did not control NOx intake [14,17]. Onur and colleagues showed that patients with RA had higher serum NOx levels than controls, and that NOx was correlated significantly with C-reactive protein and clinical disease activity [18]. The subjects were told to avoid foods high in nitrate for 3 days before blood samples were taken, and were asked to fast overnight before sampling blood [18]. Pham and colleagues showed that patients with RA had significantly higher serum NOx levels than normal control individuals and patients with OA [19].

The investigations of Onur and colleagues and Pham and colleagues did not correct for renal function. Choi noted higher serum NOx levels in patients with RA than in healthy individuals, but he found no correlation of serum NOx with disease activity measures [20]. In his studies, subjects were fasted for only 12 hours, and there was no correction for renal function. Ersoy and colleagues noted that patients with RA had higher serum NOx levels than normal controls, and that NOx levels were significantly correlated with disease activity [21]. These investigators did not control NOx intake or correct for renal function. Studies with stable isotopic (non-radioactive) L-arginine can be useful in the evaluation of NO formation *in vivo *in humans [44]. However, renal function abnormalities can result in difficulties in the interpreting results, because serum and urine nitrate and nitrite containing the isotope label are the measured products.

Our study of patients with RA with normal renal creatinine clearance illustrates that serum NOx and urine NOx excretion levels may be difficult to use as measures of whole-body NO production, even when one carefully controls NOx ingestion. We found under conditions of stringent dietary control that urinary NOx levels are no higher in patients with RA than in controls. NO in the presence of superoxide can produce peroxynitrite, which in turn can nitrate the phenolic group of amino acids (especially tyrosine) [23]. Nitrotyrosines were not assessed in the present study, but others have previously detected nitrotyrosine in serum of patients with active RA [16]. Although NO incorporated into nitrotyrosines may represent a source of unmeasured NO catabolite, it is unlikely to be more than 10% of the total NO formed in the body [45].

Urinary NOx has been generally accepted as a measure of total body NO production [10,12,46-49]. The proportion of NOx excreted in the urine constitutes 50 to 60% of the total body clearance of these compounds, with the remaining amounts being eliminated in unknown proportions by the exocrine glands and by the respiratory and gastrointestinal tracts [11,12]. The total amount of NOx measured in the urine and serum derives from both endogenous and exogenous sources. As shown in our study, individuals vary substantially in their dietary intake of nitrate and nitrite. Unless individual variability of NOx intake is taken into account, measures of NOx in urine and serum will probably not reflect endogenous NO production, and exogenous NOx might obscure true differences in endogenous NOx production. Grabowski and colleagues [15] found in healthy volunteers that urinary NOx levels after an overnight fast are not significantly altered by dietary intake of nitrate and nitrite. Our results demonstrate a significant decrease in NOx in urine and serum after 24 hours of a NOx-restricted diet. However, we do not demonstrate a significant difference in NOx in urine between normal subjects and patients with RA. This finding suggests that the patients with RA may have differed from control subjects in the renal elimination of NOx. Grabowski and colleagues [15] noted a higher average urinary nitrate/creatinine ratio in patients with RA than in controls after only an overnight fast. Our analyses showed that urinary NOx and NOx/creatinine levels after 1, 2, or 3 days of a NOx-restricted diet were not different between normal and RA subjects, but serum NOx/creatinine levels were significantly higher in our study in patients with RA. However, this difference is difficult to interpret in view of the finding that patients with RA have a decrease in renal NOx clearance.

Nitrite and nitrate are each filtered by the kidney and reabsorbed in the proximal tubule, but there may be other sites of tubular reabsorption and secretion [50]. Altered renal NOx clearance has not previously been described in humans except in cases with substantial reductions of glomerular filtration [51]. In the present study, the RA and control groups had similar creatinine clearances, and none of the subjects had a serum creatinine above 2 mg/dl. Thus, differences in glomerular filtration do not seem to explain the lower NOx clearances in the RA group. Moreover, treatment with NSAIDs, prednisone, or methotrexate was not associated with a lower NOx clearance, pointing away from concomitant anti-rheumatic therapy as the cause of the reduced NOx clearance. This analysis must be interpreted with caution in view of the small numbers of subjects in each of the medication subgroups and the fact that many of the patients were taking multiple anti-rheumatic agents. We suspect that the altered renal clearances and fractional excretions of NOx in patients with RA are due to intrinsic renal tubular abnormalities. Renal tubular abnormalities have been described in patients with RA and in those with Sjögren's syndrome [52,53].

Exocrine dysfunction of the lacrimal and salivary glands may occur in RA, leading in some cases to secondary Sjögren's syndrome [35-39]. Thus, alterations in NOx excretion (and possibly NOx clearance comparable to those we note in the kidney) by the salivary glands might influence NOx levels in serum and urine. Our studies document low basal salivary flow and tear flow in patients with RA, but salivary NOx levels after 3 days of a low NOx diet were comparable in controls and in subjects with RA. Although salivary flow can be influenced by NO [31], we find no evidence that this is disordered in RA. We did not measure NOx clearance by the salivary glands. We speculate that salivary gland epithelium, like renal tubular cell function, which we showed here has an altered NOx clearance in RA, may also have diminished NOx clearance. This could modify the excretion of NOx into saliva and partly contribute to the elevated serum NOx in RA.

Our earlier report of studies of these subjects showed that NOS activity of freshly isolated PBMCs was significantly higher in subjects with RA and that isolated PBMCs produced more NOx *in vitro *in the basal state and after stimulation with IFN-γ. In addition, NOS activity was significantly correlated with the number of swollen and tender joints [22]. Although NOx is much easier to measure than NOS activity, our results emphasize the limitations of using serum and urine NOx levels as indices of NOS activity and NO production in patients with RA. We have shown how dietary influences and altered urinary NOx clearances make it difficult to interpret serum and urine NOx levels. Future studies of NO production in RA are therefore likely to be more informative if they focus on specific anatomic or cellular compartments relevant to the pathophysiology of disease.

## Conclusion

Subjects with RA may have altered renal clearance and fractional excretion of NOx. This complicates the interpretation of measures of serum NOx concentrations and urine NOx levels, even when one carefully controls NOx ingestion. NOx measures as parameters of RA activity must be used with caution. Studies of NO production in RA are likely to be more informative if they focus on specific anatomic or cellular compartments relevant to the pathophysiology of disease.

## Abbreviations

DUMC = Duke University Medical Center; IFN = interferon; IL = interleukin; mHAQ = Modified Stanford Health Assessment Questionnaire; NO = nitric oxide; NOS = nitric oxide synthase; NOS2 = inducible nitric oxide synthase; NOx = nitrite + nitrate; NSAID = nonsteroidal anti-inflammatory drug; OA = osteoarthritis; PBMC = peripheral blood mononuclear cell; RA = rheumatoid arthritis; TNF = tumor necrosis factor.

## Competing interests

The authors declare that they have no competing interests.

## Authors' contributions

JBW, WEW, DSP, and EWS planned the overall study. JBW supervised the study and laboratory analyses. TL and EWS recruited and examined patients and controls, and collected clinical information. WEW performed the statistical analyses. JBW wrote the manuscript, and TL, WEW, DSP, and EWS edited the manuscript. All authors read and approved the final manuscript.

## Figures and Tables

**Table 1:** Oral NOx intake before admission and during ingestion of the nitrate- and nitrite-restricted diet

Period	Group	*n*	Median NOx intake (μmol) (interquartile range)	*p*
Before admission	Normal	20	1,875 (94–13,900)	NS
	RA	25	957 (101–5,162)	
Hospital day 1 (0–24 h)	Normal	20	111 (98–139)	
	RA	25	98 (54–150)	
Hospital day 2 (24–48 h)	Normal	20	126 (98–140)	
	RA	25	123 (89–140)	
Hospital day 3 (48–72 h)	Normal	20	126 (112–140)	
	RA	25	119 (76–140)	

NOx, nitrite + nitrate; NS, not significant (comparing normal and RA); RA, rheumatoid arthritis.

**Table 2:** Renal clearance of creatinine and NOx, and fractional excretion of NOx

Measure	Group	*n*	Median (interquartile range)	*p*
Creatinine clearance (ml/min)	Normal	20	84 (74–97)	NS (0.071)
	RA	25	99 (82–133)	
NOx clearance (ml/min), 0–24 h	Normal	17	125(72–209)	0.004
	RA	23	31 (16–73)	
NOx clearance (ml/min), 24–48 h	Normal	17	69 (38–106)	0.026
	RA	23	38 (20–58)	
NOx clearance (ml/min), 48–72 h	Normal	17	47 (20–80)	0.014
	RA	23	27 (11–34)	
NOx fractional excretion, 0–24 h	Normal	17	1.40 (0.78–2.26)	0.004
	RA	23	0.35 (0.19–0.82)	
NOx fractional excretion, 24–48 h	Normal	17	0.81 (0.48–1.23)	0.010
	RA)	23	0.29 (0.17–0.71)	
NOx fractional excretion, 48–72 h	Normal	17	0.56 (0.16–0.94)	0.010
	RA	23	0.28 (0.11–0.38)	

NOx, nitrite + nitrate; NS, not significant (comparing normal and RA); RA, rheumatoid arthritis.

**Table 3:** Saliva and lacrimal measures

Measure	Group	*n*	Median (interquartile range)	*p*^a^
Unstimulated salivary flow (ml/min)	Normal	20	5.5 (4.0–9.5)	0.0009
	RA	25	3.0 (1.0–5.0)	
Stimulated salivary flow, 15 min (ml/min)	Normal	20	8.0 (3.8–10.5)	0.0005
	RA	25	4.0 (1.5–4.5)	
Basal salivary NOx (μM)^b^	Normal	20	46.1 (19.5–71.3)	NS
	RA	25	48.8 (31.3–83.4)	
Stimulated salivary NOx, 15 min (μM)^b^	Normal	20	48.1 (26.6–98.7)	NS
	RA	25	37.0 (15.7–81.9)	
Schirmer's tear test, left (mm)	Normal	19	14.0 (5.0–30.0)	0.025
	RA	25	8.0 (2.0–17.5)	
Schirmer's tear test, right (mm)	Normal	19	12.0 (5.0–30.0)	0.008
	RA	25	5.0 (1.5–10.0)	

NOx, nitrite + nitrate; ^a^NS*, not significant; RA, rheumatoid arthritis. ^a^Comparing normal and RA; ^b^saliva collected on the third day of the diet.

## References

[B1] Abramson SB, Amin AR, Clancy RM, Attur M (2001). The role of nitric oxide in tissue destruction. Best Pract Res Clin Rheumatol.

[B2] Wallace JL (2005). Nitric oxide as a regulator of inflammatory processes. Mem Inst Oswaldo Cruz.

[B3] Ialenti A, Moncada S, Di Rosa M (1993). Modulation of adjuvantarthritis by endogenous nitric oxide. Br J Pharmacol.

[B4] Stefanovic-Racic M, Meyers K, Meschter C, Coffey JW, Hoffman RA, Evans CH (1994). N-monomethyl arginine, an inhibitor of nitric oxide synthase, suppresses the development of adjuvant arthritis in rats. Arthritis Rheum.

[B5] McCartney-Francis N, Allen JB, Mizel DE, Albina JE, Xie QW, Nathan CF, Wahl SM (1993). Suppression of arthritis by an inhibitor of nitric oxide synthase. J Exp Med.

[B6] Connor JR, Manning PT, Settle SL, Moore WM, Jerome GM, Webber RK, Tjoeng FS, Currie MG (1995). Suppression of adjuvant-induced arthritis by selective inhibition of inducible nitric oxide synthase. Eur J Pharmacol.

[B7] Weinberg JB, Granger DL, Pisetsky DS, Seldin MF, Misukonis MA, Mason SN, Pippen AM, Ruiz P, Wood ER, Gilkeson GS (1994). The role of nitric oxide in the pathogenesis of spontaneous murine autoimmune disease: increased nitric oxide production and nitric oxide synthase expression in MRL-*lpr*/*lpr *mice, and reduction of spontaneous glomerulonephritis and arthritis by orally administered *N*^G^-monomethyl-l-arginine. J Exp Med.

[B8] McCartney-Francis NL, Song XY, Mizel DE, Wahl CL, Wahl SM (1999). Hemoglobin protects from streptococcal cell wall-induced arthritis. Arthritis Rheum.

[B9] Weinberg JB, Laskin J, Laskin DL (1999). Nitric oxide and inflammation in autoimmune disorders. Cellular and Molecular Biology of Nitric Oxide.

[B10] Granger DL, Anstey NM, Miller WC, Weinberg JB (1999). Measuring nitric oxide production in human clinical studies. Methods Enzymol.

[B11] Green LC, de Luzuriaga KR, Wagner DA, Rand W, Istfan N, Young VR, Tannenbaum SR (1981). Nitrate biosynthesis in man. Proc Natl Acad Sci USA.

[B12] Jungersten L, Edlund A, Petersson AS, Wennmalm A (1996). Plasmanitrate as an index of nitric oxide formation in man – analyses of kinetics and confounding factors. Clin Physiol.

[B13] Lundberg JO, Govoni M (2004). Inorganic nitrate is a possible source for systemic generation of nitric oxide. Free Radic Biol Med.

[B14] Ueki Y, Miyake S, Tominaga Y, Eguchi K (1996). Increased nitric oxide levels in patients with rheumatoid arthritis. J Rheumatol.

[B15] Grabowski PS, England AJ, Dykhuizen R, Copland M, Benjamin N, Reid DM, Ralston SH (1996). Elevated nitric oxide production in rheumatoidarthritis – detection using the fasting urinary nitrate/creatinine ratio. Arthritis Rheum.

[B16] Kaur H, Halliwell B (1994). Evidence for nitric oxide-mediated oxidative damage in chronic inflammation – nitrotyrosine in serum and synovial fluid from rheumatoid patients. FEBS Lett.

[B17] Farrell AJ, Blake DR, Palmer RM, Moncada S (1992). Increased concentrations of nitrite in synovial fluid and serum samples suggest increased nitric oxide synthesis in rheumatic diseases. Ann Rheum Dis.

[B18] Onur O, Akinci AS, Akbiyik F, Unsal I (2001). Elevated levels of nitrate in rheumatoid arthritis. Rheumatol Int.

[B19] Pham TN, Rahman P, Tobin YM, Khraishi MM, Hamilton SF, Alderdice C, Richardson VJ (2003). Elevated serum nitric oxide levels in patients with inflammatory arthritis associated with co-expression of inducible nitric oxide synthase and protein kinase C-ε in peripheral blood monocyte-derived macrophages. J Rheumatol.

[B20] Choi JW (2003). Nitric oxide production is increased in patients with rheumatoid arthritis but does not correlate with laboratory parameters of disease activity. Clin Chim Acta.

[B21] Ersoy Y, Ozerol E, Baysal O, Temel I, MacWalter RS, Meral U, Altay ZE (2002). Serum nitrate and nitrite levels in patients with rheumatoid arthritis, ankylosing spondylitis, and osteoarthritis. Ann Rheum Dis.

[B22] St Clair EW, Wilkinson WE, Lang T, Sanders L, Misukonis MA, Gilkeson GS, Pisetsky DS, Granger DL, Weinberg JB (1996). Increased expression of blood mononuclear cell nitric oxide synthase type 2 in rheumatoid arthritis patients. J Exp Med.

[B23] Beckman JS, Chen J, Ischiropoulos H, Crow JP (1994). Oxidative chemistry of peroxynitrite. Methods Enzymol.

[B24] Arnett FC, Edworthy SM, Bloch DA, McShane DJ, Fries JF, Cooper NS, Healey LA, Kaplan SR, Liang MH, Luthra HS (1988). The American Rheumatism Association 1987 revised criteria for the classification of rheumatoid arthritis. Arthritis Rheum.

[B25] Perkins DJ, St Clair EW, Misukonis MA, Weinberg JB (1998). Reduction of NOS2 overexpression in rheumatoid arthritis patients treated with anti-tumor necrosis factor α monoclonal antibody (cA2). Arthritis Rheum.

[B26] Pincus T, Summey JA, Soraci SA, Wallston KA, Hummon NP (1983). Assessment of patient satisfaction in activities of daily living using a modified Stanford Health Assessment Questionnaire. Arthritis Rheum.

[B27] Hochberg M, Chang RW, Dwosh I, Lindsey S, Pincus T, Wolfe F (1992). The American College of Rheumatology 1991 revised criteria for the classification of global functional status in rheumatoid arthritis. Arthritis Rheum.

[B28] Meah MN, Harrison N, Davies A (1994). Nitrate and nitrite in foods and the diet. Food Addit Contam.

[B29] Tarpey MM, Wink DA, Grisham MB (2004). Methods for detection of reactive metabolites of oxygen and nitrogen: *in vitro* and *in vivo* considerations. Am J Physiol Regul Integr Comp Physiol.

[B30] Granger DL, Anstey AM, Miller WC, Weinberg JB (1999). Measuring nitric oxide production in human clinical studies. Methods Enzymol.

[B31] Bodis S, Haregewoin A (1993). Evidence for the release and possible neural regulation of nitric oxide in human saliva. Biochem Biophys Res Commun.

[B32] van Bijsterveld OP (1969). Diagnostic tests in the Sicca syndrome. Arch Ophthalmol.

[B33] Granger DL, Hibbs J, Broadnax LM (1991). Urinary nitrate excretion in relation to murine macrophage activation. Influence of dietary L-arginine and oral *N*^G^-monomethyl-L-arginine. J Immunol.

[B34] Pannala AS, Mani AR, Spencer JP, Skinner V, Bruckdorfer KR, Moore KP, Rice-Evans CA (2003). The effect of dietary nitrate on salivary, plasma, and urinary nitrate metabolism in humans. Free Radic Biol Med.

[B35] Andonopoulos AP, Drosos AA, Skopouli FN, Moutsopoulos HM (1989). Sjogren's syndrome in rheumatoid arthritis and progressivesystemic sclerosis. A comparative study. Clin Exp Rheumatol.

[B36] Averns H, Hall J, Webley M (1999). Role of opticare eye drop delivery system in patients with rheumatoid arthritis. J Rheumatol.

[B37] Jensen JL, Uhlig T, Kvien TK, Axell T (1997). Characteristics of rheumatoid arthritis patients with self-reported sicca symptoms: evaluation of medical, salivary and oral parameters. Oral Dis.

[B38] Mody GM, Hill JC, Meyers OL (1988). Keratoconjunctivitis sicca in rheumatoid arthritis. Clin Rheumatol.

[B39] Brun JG, Madland TM, Jonsson R (2003). A prospective study of sicca symptoms in patients with rheumatoid arthritis. Arthritis Rheum.

[B40] Forstermann U, Kleinert H, Gash I, Schwarz P, Closs EI, Dun NJ (1995). Expression and expressional control of nitric oxide synthases in various cell types. Adv Pharmacol.

[B41] Weinberg JB (1998). Nitric oxide production and nitric oxide synthase type 2 expression by human mononuclear phagocytes – a review. Mol Med.

[B42] Weinberg JB (1998). Nitric oxide as an inflammatory mediator in autoimmune MRL-*lpr*/*lpr *mice. Environ Health Perspect.

[B43] Sakurai H, Kohsaka H, Liu MF, Higashiyama H, Hirata Y, Kanno K, Saito I, Miyasaka N (1995). Nitric oxide production and inducible nitric oxide synthase expression in inflammatory arthritides. J Clin Invest.

[B44] Castillo L, Beaumier L, Ajami AM, Young VR (1996). Whole body nitric oxide synthesis in healthy men determined from [^15^N]arginine-to-[^15^N]citrulline labeling. Proc Natl Acad Sci USA.

[B45] Beckman JS (1996). Oxidative damage and tyrosine nitration from peroxynitrite. Chem Res Toxicol.

[B46] Granger DL, Miller WC, Hibbs JB, Feelisch M, Stamler JS (1996). Methods of analyzing nitric oxide production in the immune response. Methods in Nitric Oxide Research.

[B47] Granger DL, Taintor RR, Boockvar KS, Hibbs JB (1995). Determination of nitrate and nitrite in biological samples using bacterial nitrate reductase coupled with the Griess reaction. Methods.

[B48] Suzuki H, Ikenaga H, Hishikawa K, Nakaki T, Kato R, Surata T (1992). Increases in nitrite/nitrate excretion in the urine as an indicator of the release of endothelium-derived relaxing factor during elevation of blood pressure. Clin Sci.

[B49] Bode-Böger SM, Böger HR, Creutzig A, Tsikas D, Gutzki F-M, Alexander K, Frölich JC (1994). L-arginine infusion decreases peripheral arterial resistance and inhibits platelet aggregation in healthy subjects. Clin Sci.

[B50] Suto T, Losonczy G, Qiu CB, Hill C, Samsell L, Ruby J, Charon N, Venuto R, Baylis C (1995). Acute changes in urinary excretion of nitrite plus nitrate do not necessarily predict renal vascular NO production. Kidney Int.

[B51] Mackenzie IMJ, Ekangaki A, Young JD, Garrard CS (1996). Effect of renal function on serum nitrogen oxide concentrations. Clin Chem.

[B52] Talal N (1971). Sjogren's syndrome, lymphoproliferation, and renal tubular acidosis. Ann Intern Med.

[B53] Niederstadt C, Happ T, Tatsis E, Schnabel A, Steinhoff J (1999). Glomerular and tubular proteinuria as markers of nephropathy in rheumatoid arthritis. Rheumatology (Oxford).

